# Quantifying the Computational Efficiency of Compressive Sensing in Smart Water Network Infrastructures

**DOI:** 10.3390/s20113299

**Published:** 2020-06-10

**Authors:** George Tzagkarakis, Pavlos Charalampidis, Stylianos Roubakis, Antonis Makrogiannakis, Panagiotis Tsakalides

**Affiliations:** 1 Institute of Computer Science, Foundation for Research and Technology-Hellas, GR70013 Heraklion, Greece; pcharala@ics.forth.gr (P.C.); roub@ics.forth.gr (S.R.); makrog@ics.forth.gr (A.M.); tsakalid@ics.forth.gr (P.T.); 2 Department of Computer Science, University of Crete, GR70013 Heraklion, Greece

**Keywords:** smart water networks, Internet-of-Things platform, compressive sensing, energy consumption, execution speedup, weak encryption

## Abstract

Monitoring contemporary water distribution networks (WDN) relies increasingly on smart metering technologies and wireless sensor network infrastructures. Smart meters and sensor nodes are deployed to capture and transfer information from the WDN to a control center for further analysis. Due to difficulties in accessing the water assets, many water utility companies employ battery-powered nodes, which restricts the use of high sampling rates, thus limiting the knowledge we can extract from the recorder data. To mitigate this issue, compressive sensing (CS) has been introduced as a powerful framework for reducing dramatically the required bandwidth and storage resources, without diminishing the meaningful information content. Despite its well-established and mathematically rigorous foundations, most of the focus is given on the algorithmic perspective, while the real benefits of CS in practical scenarios are still underexplored. To address this problem, this work investigates the advantages of a CS-based implementation on real sensing devices utilized in smart water networks, in terms of execution speedup and reduced ener experimental evaluation revealed that a CS-based scheme can reduce compression execution times around 50%, while achieving significant energy savings compared to lossless compression, by selecting a high compression ratio, without compromising reconstruction fidelity. Most importantly, the above significant savings are achieved by simultaneously enabling a weak encryption of the recorded data without the need for additional encryption hardware or software components.

## 1. Introduction

Drinking water supplies face pressing issues, particularly in island regions, where climate change, water scarcity, pollution, and the high cost of desalination are putting pressure on water distribution organisations. At the same time, 15–25% of the drinking water produced is lost via invisible leakages, which represent a main contributor to non-revenue water. From an economic perspective, the cost of lost water worldwide, due to leakages, metering errors and non-billed consumption, is about US$39 billion annually [1]. In addition, the volume and indicators of non-revenue water vary with variations in the system input volume, which is even more critical for monitoring non-revenue water for systems alternating between intermittent and continuous supply [2]. The challenge for water utility companies is to save resources, thus improving water sustainability. To this end, innovative monitoring and control technologies to reduce water loss are increasingly gaining the interest of the water management communities.

Specifically, water utility companies progressively transform their obsolete water distribution networks (WDNs) to smart infrastructures by exploiting modern Information Communication Technologies (ICT) [3]. A smart water network infrastructure aims at reducing telemetry costs, detecting leakages in a timely fashion, monitoring the non-revenue water, and visualizing the available data in a user-friendly way. A typical architecture of such an infrastructure consists of two main entities, depending on the functionalities they offer and their spatial location in the space, namely, at the edges of the network (i.e., the hydraulic network of pipes, the consumers’ buildings, etc.) and in the control center [4]. Our subsequent analysis focuses on the edges of the water network, since the need to satisfy any energy and computational constraints mainly refers to this specific part of the architecture.

In the literature, we often find an additional partition at the edges of the network, namely between a physical infrastructure and the smart sensors [5,6,7]. Specifically, the physical infrastructure includes the tanks where the water is stored, the pipes through which the water is distributed to the network, joints for pipes connection, and water meters to record consumption. Systematic recording of the technical characteristics of the physical infrastructure is imperative, since they affect the hydraulic models and processing of the observed data. Such parameters include, among others, the pipes’ length, diameter and roughness; pump curves and settings; and the number of tanks and their dimensions [8]. On the other hand, the smart sensors are the part of the architecture associated with the collection, processing, and transmission of the relevant data. These sensors are utilized to control water quality, pressure, and flow, while modern smart meters also provide leakage detection and notification capabilities. In addition, the smart sensing infrastructure also includes all the appropriate network components, such as transmitters and gateways, to send the observed data to a control center for further analysis.

Smart sensing technologies are based on the development of smart metering and sensing devices [9,10], in conjunction with advanced numerical methods for high-level data analysis [11,12,13]. Compared to traditional metering devices, smart metering deployments are a key component for the realization of smart environments, since they enable multiple capabilities to water utility companies and consumers, such as accurate data collection, backflow measurements, which is widely used problem indicator in water systems, while they are less susceptible to corrosion. The data collected from a smart infrastructure enables to better comprehend water demands, which further influences the efficient design of urban water supply networks.

Recording and analyzing data in real time allows water utilities to perform various critical tasks, such as identifying leakages, fixing system’s malfunctions, timely scheduling infrastructure maintenance, and essentially enabling them to achieve sustainable water use [14]. To this end, existing water management systems primarily rely on energy consuming above-ground deployments to monitor and transmit water network states, such as water flow and pressure, to a server periodically, typically via the mobile cellular networks, in order to detect abnormal events such as water leakages and bursts [15,16,17]. Nevertheless, more than 97% of water network assets are placed at a considerable distance from power resources and often in geographically remote areas. Such constraints put big challenges on current approaches making them unsuitable for next generation smart water networks.

To overcome these limitations, traditional water metering devices of mechanical type, are gradually replaced by sophisticated battery-driven wireless sensor networks, which are emerging as an effective alternative solution for large-scale smart water management systems [18]. However, the main challenge of these infrastructures is that sensor nodes typically consume a lot of energy to record and transmit high-precision data [19]. This constraint limits the amounts of data that can be sensed and relayed for analysis, which is necessary for timely and reliable anomaly detection (e.g., leakages and bursts) and alerting. To address this problem, reducing the volume of data that are transmitted to a control center for further processing is a critical task. To this end, data compression mechanisms are integrated on the sensor network’s side. The role of data compression in WDN management is twofold: (i) increase the system’s autonomy by reducing the energy consumption; and (ii) reduce telemetry costs for the water utility companies. To this end, reduction of data volumes is achieved by algorithms roughly classified into: (a) lossless compression methods, when perfect data reproduction is required; and (b) lossy compression methods, when perfect reproduction is either impossible or requires too many bits [20].

Each compression method has its own advantages and limitations. Specifically, in lossless compression [21,22], the recorded data stream can be reconstructed completely without losing information, while lossy compression [23,24,25] introduces a reconstruction error. Nevertheless, in contrast to lossless compression, which places an upper bound on the compression performance, lossy compression can significantly reduce the amount of data, and consequently the communication cost without sacrificing the meaningful information content.

Focusing on time series data, existing lossless and lossy compression methods are applied primarily on temporal samples, with the sampling process being largely dominated by the traditional Nyquist–Shannon theory. According to this theory, the exact recovery of a discrete signal requires a sampling rate twice the signal’s bandwidth. Moreover, the sampling scheme characteristics can have dramatic consequences on the quality of the recorded signals, the hardware necessary to achieve the required quality and therefore the cost, time, and effort that accompany the process. Nevertheless, several studies have shown that many natural signals are amenable to highly sparse representations in appropriate transform domains (e.g., wavelets and sinusoids) [26,27]. This means that the resulting vector of transform coefficients has a small number of significant (i.e., large-amplitude) elements, while the great majority of them have an amplitude equal to or near zero.

Compressive sensing (CS) provides a powerful framework for simultaneous sensing and compression [28,29], enabling a significant reduction in the sampling, computation, and transmission costs on a sensor node with limited memory and power resources. According to the theory of CS, a signal having a sparse representation in a suitable transform basis can be reconstructed from a small set of projections onto a second, measurement basis that is incoherent with the first one. Intuitively, this means that the vectors of the measurement basis are not statistically correlated with the vectors of the sparsity basis. In the framework of smart water networks, the advantages of CS have recently been exploited for reducing the amount of transmitted pressure data, thus extending the battery life of sensor nodes deployed in a WDN demonstrator [30], while still maximizing the received information to data centers. Nevertheless, this study was performed in a rather ex post fashion, in the sense that the principles of CS were applied on the recorded full-resolution time series under simulated sensing and water network conditions. Despite the well-established and mathematically rigorous foundations of CS, most of the focus is given on the algorithmic perspective, while the real benefits of CS in practical scenarios are still underexplored.

To address this problem, this work investigates the advantages of implementing a CS mechanism for lossy data compression on real sensing devices utilized in a real urban WDN, in terms of execution speedup and reduced energy consumption, when compared against a lossless compression alternative that is widely used in commercial hardware solutions. It is also important to emphasize that a water management system is required to manage confidential data, such as household consumption. Traditional systems employ a separate software- or hardware-based component to encrypt sensitive data, which increases the deployment cost of the overall infrastructure. In this work, we also demonstrate the efficiency of CS as an effective mechanism for simultaneous data compression and weak encryption, ensuring data confidentiality with high probability, in real smart water network scenarios.

The rest of the paper is organized as follows. Section 2 overviews the basic concepts of CS for data compression, and describes our CS-based system architecture enabling weak data encryption. Section 3 analyzes the complete hardware and software platform, which is utilized to quantify the efficiency of CS in a real setting. In Section 4, the performance of CS is evaluated and compared against lossless compression on real pressure data. Section 5 summarizes the main results and proposes directions for further research.

## 2. Data Acquisition System Overview and Preliminaries

Municipalities and water utility companies cannot easily afford the reduced efficiency of obsolete water distribution networks, the increased cost of maintenance and energy consumption, and the lower revenues that it generates. To overcome these limitations, water utilities show an ever increasing interest in deploying innovative sensor nodes and smart meters to bring outdated water networks to the smart water networks’ era. All these smart devices transmit consumption, flow, and pressure data periodically in predetermined time intervals, through mobile cellular networks or the Internet to a central server for further data analysis and decision making. This is also the case in our parent project, SmartWater2020 (https://www.smartwater2020.eu), which focuses on developing smart technologies to support water utilities in the islands of Crete (Greece) and Cyprus. Specifically, this study employed pressure data from a real operational network in the region of Malevizi in Crete, to evaluate and assess the operational benefits of compressive sensing in real-world scenarios. The rest of this section describes our data acquisition and compression system, and overviews the main principles and properties of compressive sensing.

### 2.1. Data Description

**Figure 1:**
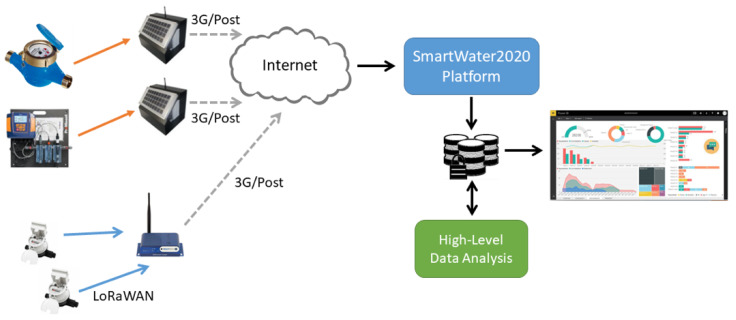
The SmartWater2020 platform.

The subsequent performance evaluation utilized real pressure data recorded by the smart water management infrastructure (see Figure 1) of the Municipal Enterprise for Water Supply and Sewerage of Malevizi. Part of the infrastructure, including smart meters and pressure sensors, has been upgraded within the framework of the SmartWater2020 project. More specifically, our dataset consists of pressure data spanning the period between January 2017 and September 2018. Incoming and outgoing flow pressure measurements are sent to the control center at a frequency of one sample (pressure expressed in Bars) per 15 min, yielding a total of approximately 9000 observations per sensor. This sampling frequency suffices in order to enable real-time monitoring of the water distribution network of the municipality of Malevizi, which is divided into 10 zones, each monitored by a pressure sensor. It is also important to emphasize that, due to the complex mountainous topography of the monitored area, the pressure time series are characterized by distinct dynamics. This allows us to demonstrate the efficiency of our CS-based system under non-uniform conditions of the WDN. We also emphasize that, although our evaluation was performed on pressure data, the same analysis could be applied in a straightforward fashion on any other type of sensed parameters, such as flow and water quality (e.g., pH, conductivity, turbidity, etc.).

### 2.2. Compressive Sensing

A critical issue in smart water networks is the increase of their autonomy by reducing the energy consumption for sampling, compressing, and transmitting the observed data. For decades, traditional signal processing methods have been largely dominated by the well-established Nyquist–Shannon sampling theorem, which states that the exact recovery of a discrete signal requires a sampling rate at least twice the highest frequency occurring in the signal. Compressive sensing (CS) [28,29] emerged as a powerful framework for simultaneous sensing and compression, enabling a significant reduction in sampling and computation costs for sensor nodes with limited memory and power resources. According to the CS theory, a signal having a sparse representation in a suitable transform basis can be reconstructed accurately from a small set of random projections, the so-called measurements, onto a second, measurement basis that is incoherent with the transform one. CS-enabled compression is achieved by the fact that the number of generated measurements is much smaller than the number of the recorded signal samples. A key property of CS is the asymmetrical computational complexity of the compression process, with the low-complexity compression stage consisting of simple linear projections, while the main computational burden is on the decompression part, taking place at a control center, where increased computational and power resources are available.

Doing so, a typical CS-based system consists of two distinct modules according to the functionality they perform. The compression module (or encoder) is responsible for generating the reduced set of random measurements from the observed data. The reduction in data volume to be transmitted yields an increased autonomy of the remote monitoring infrastructure, while reducing telemetry costs for the water utility. On the other hand, the decompression module (or decoder) reconstructs the original signal from the received set of random measurements. These two modules are further analyzed in the following sections.

#### 2.2.1. CS-Based Compression

More specifically, let $\Psi \in R^{N\times P}$ be a matrix whose columns correspond to a possibly overcomplete (i.e., N<P) transform basis. Overcompleteness ensures a more stable, robust, or compact decomposition than using a conventional basis [31]. Let $x\in R^{N}$ be an observed discrete-time signal of *N* samples, which is associated to a transform coefficients’ vector $\alpha \in R^{P}$ over the basis Ψ, as follows,

(1) α=Ψx.

**Figure 2:**
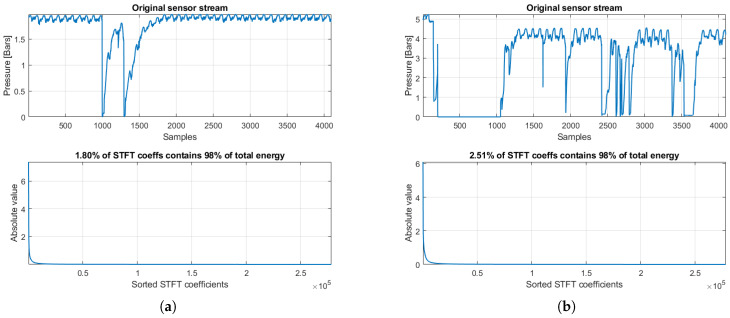
(**a**) Original pressure stream and sorted absolute STFT coefficients, under normal network conditions; and (**b**) original pressure stream and sorted absolute STFT coefficients, under abnormal network conditions.

Then, x is said to be *S*-sparse, with S<N, in basis Ψ, if α has only *S* nonzero elements. In practice, only a few signals are truly sparse, but instead they are compressible. A signal x is compressible in basis Ψ if the magnitudes of the sorted transform coefficients decay rapidly following a power law, that is,

(2) $$|\alpha_{s}|\leq Cs^{-q},\;s=1,2,\ldots ,P\;,$$

where *C* and *q* are positive constants. The larger is the *q*, the faster is the magnitude’s decay, and the more compressible is the signal. Typical examples of sparsifying transformations that have been proven efficient for a broad range of natural signals include the short-time Fourier transform (STFT), the discrete cosine transform (DCT), and the discrete wavelet transform (DWT) with its variants [27]. Without loss of generality, in the subsequent analysis, we employ the STFT, which showed a good trade-off between the achieved compressibility of our pressure signals and the required reconstruction time. Figure 2 shows two pressure streams from our dataset under normal (left) and abnormal (right) network conditions, along with the corresponding sorted absolute values of the STFT coefficients (i.e., the vector $\alpha_{s}$). Notably, in both cases, a very small percentage of the STFT coefficients, 1.80% and 2.51%, respectively, conveys 98% of the total energy of the transform coefficients α, defined by

(3) $$E_{\alpha }=\sum_{i=1}^{P}{\left|\alpha_{i}\right|}^{2}\;.$$

This reveals a high compressibility capability of the STFT for the pressure streams considered, which is aligned with the requirements of the CS framework. Nevertheless, we emphasize that the selection of the optimal sparsifying transform is beyond the scope of this study, and is left as a separate thorough analysis. Notice also that, since the stream’s reconstruction (to be analyzed in the next section), and thus the utilization of the sparsifying transform Ψ, is performed at the control center, the reconstruction time is not prohibitive for real-time scenarios given the increased computational resources therein.

In terms of signal approximation, it has been demonstrated [28] that, if a signal x is sparse or compressible in basis Ψ, then it can be reconstructed from a highly reduced set of M≪N non-adaptive linear projections, where $M=O\left(S\log\left(\frac{N}{S}\right)\right)$. From a practical perspective, instead of transmitting the originally observed *N* samples of x, a sensor reduces its consumed energy by only transmitting this significantly smaller number of *M* projections (hereafter called measurements) to the control center, where the original signal can be recovered with high accuracy for further processing.

The random measurements vector $y\in R^{M}$ is generated simply as follows,

(4) y=Φx,

where $\Phi \in R^{M\times N}$ is a measurement matrix, which must be incoherent with the sparsity basis Ψ [29]. In mathematical terms, let

(5) $$\mu \left(\Phi \Psi \right)=\max_{\begin{array}{c} i=1,\ldots ,M\\ j=1,\ldots ,P \end{array}}\left(\right|\phi_{i}^{T}\psi_{j}\left|\right)\;,$$

denote the mutual coherence between Φ and Ψ, where $\phi_{i}$ and $\psi_{j}$ are the *i*th row of Φ and *j*th column of Ψ, respectively. The parameter μ serves as a rough characterization of the degree of similarity between the sparsity and measurement systems. The smaller is the μ, the more incoherent are the two matrices. We emphasize that the data compression, which is performed onboard the sensors, consists only of a simple matrix-vector multiplication as expressed by Equation (4). The utilization of the sparsifying basis Ψ is required only during the decompression phase (see the next section), which is carried out at the control center.

Examples of measurement matrices, which are incoherent with any fixed transform basis with high probability (universality property [29]), include random matrices with independent and identically distributed (i.i.d.) Gaussian or Bernoulli entries [28], structurally random matrices [32], Toeplitz block matrices [33], and scrambled block Hadamard ensembles [34], just to name a few. Without loss of generality, in the following, we employ scrambled block Hadamard ensembles due to their computationally tractable implementation via the Fast Walsh–Hadamard Transform (FWHT) [35]. Notice that, in practice, the system operator is responsible for defining the appropriate number of measurements by setting the value of the sampling rate (SR), which is simply the ratio of the number of random measurements over the original signal length, that is, $\mathrm{SR}=\frac{M}{N}$. Given that M≪N, the computational and power savings of each sensor node stem from the fact that they process and transmit a highly compressed signal y instead of the original x. To be consistent with lossless compression, hereafter we also use compression rate (CR) instead of sample rate as the input parameter to the compressive sensing, where CR=1−SR.

#### 2.2.2. CS-Based Decompression

By employing the *M* random measurements and given the *S*-sparsity property in the transform basis, the original signal x can be recovered by taking a number of different approaches. The majority of these approaches solve constrained optimization problems, including, among others, convex relaxation [28,36] and greedy strategies [37,38]. In our implementation, the NESTA algorithm (Matlab code available at https://statweb.stanford.edu/candes/software/nesta/) [39] is employed, which is shown to achieve a good trade-off between reconstruction accuracy and computation time. We emphasize though that the scope of this paper is to illustrate the efficiency of CS in reducing compression and transmission costs, when compared against its lossless counterparts, for real sensor data recorded in water distribution networks. As such, an exhaustive comparison with the various reconstruction algorithms for finding the optimal solution is out of the scope of this study.

Focusing on the optimization problem to be solved for reconstructing the original data, NESTA solves the following synthesis-based problem,

(6) $$\min_{\alpha \in R^{P}}{∥\alpha ∥}_{1}\;s.t.\;{∥y-\Phi \left(\Psi \alpha \right)∥}_{2}<\delta \;,$$

where $\alpha \in R^{P}$ is a sparse coefficient vector, ${∥\cdot ∥}_{1}$ and ${∥\cdot ∥}_{2}$ denote the $\ell_{1}$ and $\ell_{2}$ norm, respectively, and δ>0 is a small threshold ($\delta =10^{-3}$ in our implementation). Having estimated the sparse coefficient vector, $\hat{\alpha }$, a reconstruction of the original signal is simply obtained by taking the inverse transform, that is,

(7) $$\hat{x}=\Psi^{-1}\hat{\alpha }\;.$$

As mentioned in Section 2.2.1, the short-time Fourier transform (STFT) along with scrambled block Hadamard ensembles are utilized in our CS-based system in place of the sparsifying transformation Ψ and random measurement matrix Φ, respectively. In our system, the reconstruction error is measured in terms of the signal-to-error ratio (SER) (in dB) between the original and reconstructed signals x and $\hat{x}$, respectively, defined by

(8) $$\mathrm{SER}\left(x,\hat{x}\right)=10\log_{10}\frac{{∥x∥}_{2}^{2}}{∥x-\hat{x}{∥}_{2}^{2}}\;.$$

**Figure 3:**
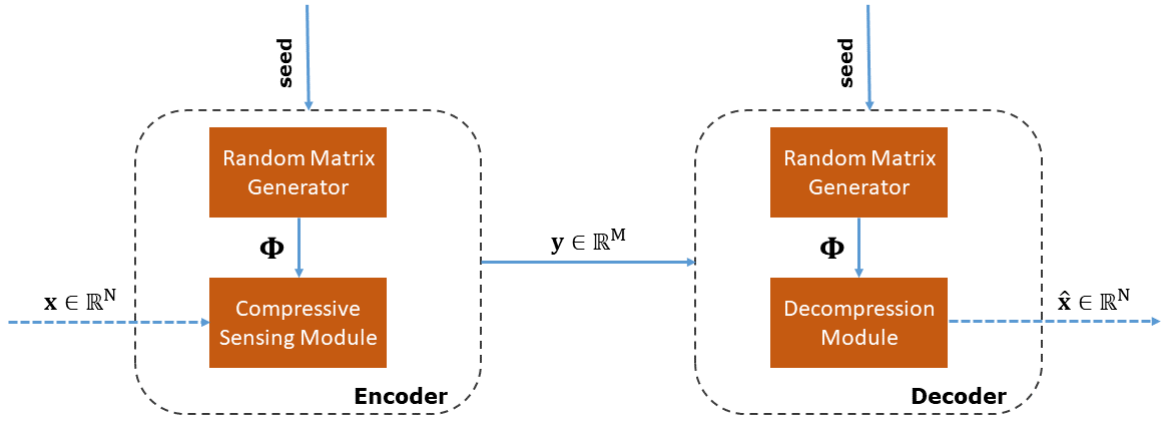
General architecture of our CS-based system.

Figure 3 summarizes the general architecture of our CS-based system. Notice that the explicit knowledge of Φ is required at the decoder side to solve the reconstruction problem. Depending on the length *N* of the original signal x and the number of measurements *M*, the size of Φ can be large enough prohibiting its transmission together with the measurements y. To alleviate this issue, only the seed (a single integer) used for generating the random measurement matrix Φ is sent to the decoder, where the pseudo-random sequence of its elements is re-generated. We emphasize that, in our system, the encoder is implemented at the edge of the water distribution network, that is, on the sensing devices. In the following, the terms compression/encoding and decompression/decoding are used interchangeably.

**Figure 4:**
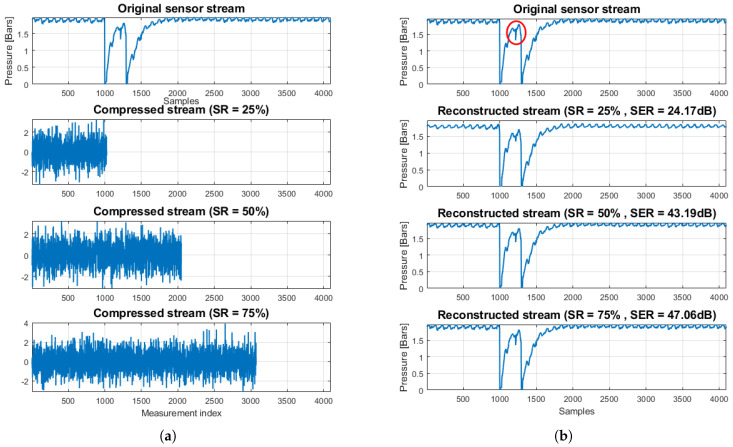
(**a**) Original stream under normal network conditions and its compressed versions; and (**b**) original stream and CS-based reconstructions, for three distinct sampling ratios SR∈{25%,50%,75%}.

As an illustration of the CS reconstruction performance, Figure 4 shows a part of an original pressure stream of a sensor from our real-world WDN, under normal network conditions. In particular, Figure 4a shows the original stream together with its compressed versions for three sampling ratios SR∈{25%,50%,75%}. It is important to emphasize that, although the original observations are pressure samples (in Bars), the compressed counterparts generated via Equation (4) are not expressed in a “physical domain”. Figure 4b shows the original along with the three reconstructed streams. As can be seen, the reconstruction quality improves as the SR increases, as expected. Most importantly, the reconstruction is already accurate enough even for SR=25%, except for some sharper details (see region in the red circles) that cannot be captured accurately when the number of random measurements *M* is small. Nevertheless, these details can be recovered very accurately as the SR increases (see SR=50%).

**Figure 5:**
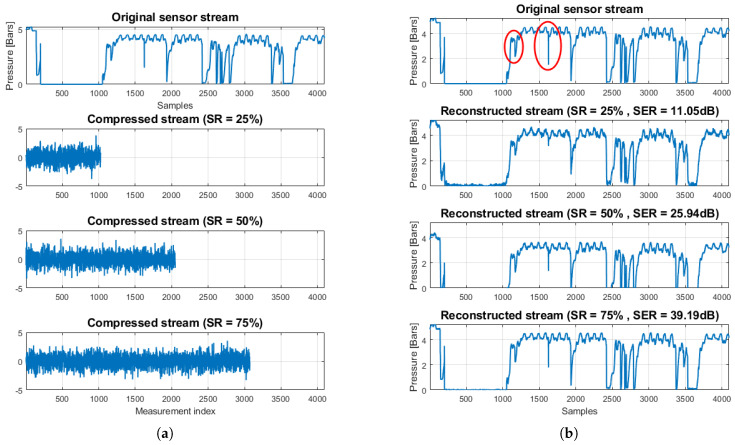
(**a**) Original stream under abnormal network conditions and its compressed versions; and (**b**) original stream and CS-based reconstructions, for three distinct sampling ratios SR∈{25%,50%,75%}.

As a second illustration, Figure 5 shows a part of an original pressure stream of a sensor from our real-world WDN, under abnormal network conditions. In particular, Figure 5a shows the original stream and its compressed versions for three sampling ratios SR∈{25%,50%,75%}. Figure 5b shows the original along with the three reconstructed streams. As in the normal case, the reconstruction quality improves as the SR increases. Most importantly, even in this case with the sharp transitions of the pressure values, the reconstruction quality is high enough even for a small sampling ratio (see SR=25%). As before, the sharp details (red circles) can be recovered very accurately as the SR increases (see SR=50%).

Most importantly, the random nature of the generated compressed measurements y, due to the random matrix Φ, results in a weak encryption property of the CS process. The encryption mechanism, which is overviewed in the next section, is inherent to the compression stage on the sensing devices, without the need for additional hardware or software components, thus reducing the deployment cost of the smart water network infrastructure.

### 2.3. CS Weak Encryption

As mentioned above, CS enables simultaneous compression and weak encryption of the observed data, without the additional computational cost of a separate cipher layer [40], for secure reception by a legitimate decoder. This is a key requirement in contemporary smart water network infrastructures, due to confidentiality and privacy issues of the recorded data. CS-based encryption is weak, in the sense that it provides computational and not perfect secrecy. In the CS case, secrecy lies in the computational difficulty in guessing the correct encryption key [41], which generates the random measurement matrix Φ, the only source of randomness in the CS process, at the decoder side.

In our case, the pseudo-random generator’s seed plays the role of the encryption key, which is sent through a secure key-exchange channel, with which the measurement matrix is recovered at the legitimate recipient [42]. The highly reduced computational and transmission costs, due to the minimal number of messages exchanged between the sender and the recipient, of this mechanism come at the cost of a critical disadvantage. Specifically, by fixing Φ between the encoder and decoder sides, the encryption process becomes deterministic. This makes the system vulnerable to an eavesdropper, who might degrade the encryption process using the method of chosen-plaintext attacks (CPA). To overcome this limitation, a compress-then-encrypt scheme was proposed by Fay and Ruland [40], where signal normalization to unit energy is performed prior to applying CS, with the signal energy being encrypted separately and sent with the compressed signal.

**Figure 6:**
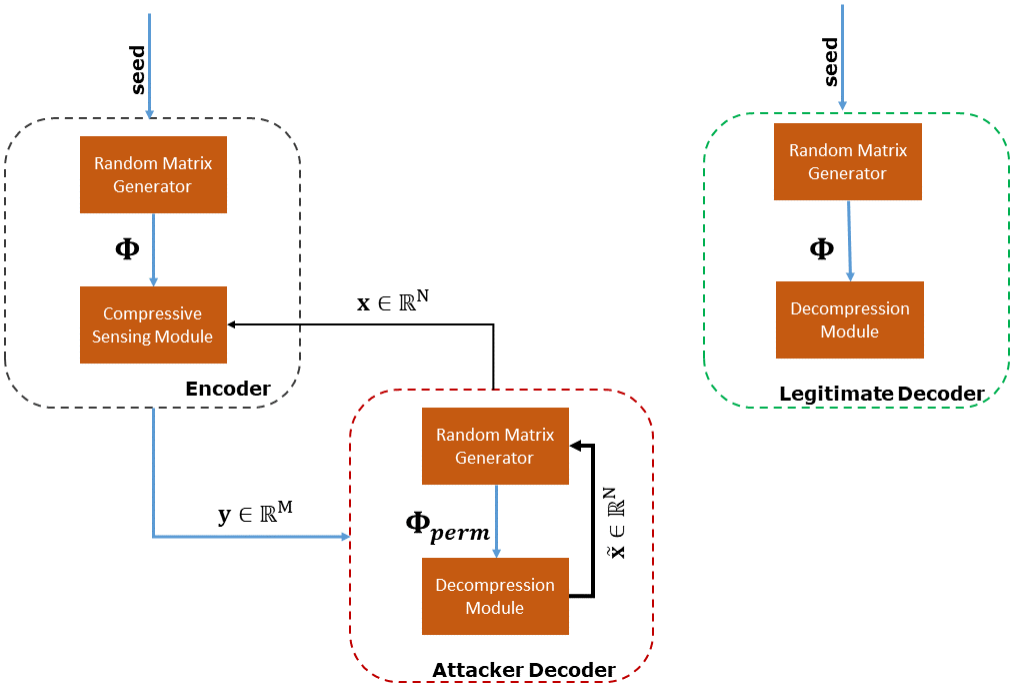
Adversarial scenario in a CS-based system.

In the subsequent analysis, we evaluate the weak encryption capability of a CS-based system by simulating the following adversarial scenario, as shown in Figure 6. Specifically, we assume that an adversary does not have access to the true original measurement matrix Φ that generated the compressed stream, but to a permutation of its rows. This scenario is simulated easily as follows,

(9) $$y_{A}=\left(P_{M}\Phi \right)x\;,$$

where $y_{A}$ are the random measurements generated by the adversary and $P_{M}\in R^{M\times M}$ is a permutation matrix which models the imperfect knowledge of the true Φ on behalf of the adversary. In the subsequent evaluation, the percentage of permuted rows of the original measurement matrix is defined by p∈[0.2,0.4,0.6,0.8,1], where, for each *p* value, ⌊p·M⌋ randomly selected rows are permuted while the remaining rows are left in the original position. When a legitimate system operator receives the compressed measurements, we assume that the permutation matrix is equivalent to the identity matrix, i.e., $P_{M}=I$.

## 3. Hardware Benchmark

To prolong the network’s lifetime, end devices of smart water networks are typically equipped with hardware that achieves low-power operation, both by minimizing the on-board components only to the bare minimum required by the application and by providing features that enable components to operate in low-power mode (e.g., MCU deep sleep and radio deactivation). It is common practice for embedded operating systems running on the devices to provide these features as energy-saving options to upper layers (i.e., applications) as well as inherently make extensive use of them [43].

Among all the operations performed on the devices, which in our case perform monitoring of a low-frequency phenomenon (water pressure), the communication task is known to be the most power-hungry, due to the high energy consumption of the radio circuitry. Thus, compression techniques employed at the application layer can offer substantial energy savings by minimizing data transmission, at the cost of additional data processing. Lossy CS-based compression, used in this work, can be tuned at a high compression rate, without compromising the decompression fidelity. Most importantly, its processing overhead is significantly smaller than the one imposed by a lossless compression alternative that is widely used in commercial hardware solutions.

A common strategy for assessing the degree to which low-power operation satisfies an application’s requirements, is to employ the so-called software-based energy profiling, performed through enabling appropriate software modules of the embedded operating system. In this section, we describe the hardware platform, software components (along with implementation details) and energy profiling tools used for assessing the energy efficiency of the proposed CS-based scheme.

### 3.1. Hardware Platform

In the following benchmarking, we use the Zolertia RE-Mote platform, an ultra-low power hardware development platform designed jointly by universities and industrial partners, in the framework of the European research project RERUM [44]. RE-Mote is a flexible platform that can support several wireless sensor networks and Internet-of-Things (IoT) applications, such as smart building automation, environmental monitoring, and Smart Cities applications. It is based on the Texas Instruments CC2538 ARM Cortex-M3 32MHz System on Chip (SoC) (https://www.ti.com/product/CC2538), with an on-board 2.4 GHz IEEE 802.15.4 Radio Frequency (RF) interface, 512KB flash memory, and 32KB RAM, as well as an 868 MHz IEEE 802.15.4 compliant RF interface (CC1200). Dual-radio support makes it suitable both for short-range/indoor and long-range/outdoor applications. Additionally, RE-Mote platform offers different interfaces (e.g., Inter-integrated Circuit (I2C), Serial Peripheral Interface (SPI), and Universal Asynchronous Receiver Transmitter (UART)) for connecting a multitude of analog and digital sensors. The platform can be battery-operated and hosts a built-in battery charger for LiPo batteries.

### 3.2. Software Description

#### 3.2.1. Contiki OS

Contiki OS is a popular open source operating system for wireless sensor networks, originally proposed in [45], which targets resource-constrained embedded devices. Recognizable for its high portability, it has been ported to several small microcontroller architectures, such as AVR, MSP430, and TI CC2538. The operating system is implemented in C programming language and uses a make/build environment for cross-compilation on most platforms. It follows an event-driven programming model along with a cooperative scheduling approach based on proto-threads [46], essentially a lightweight mechanism for pseudo-threading that helps minimizing the memory footprint of the OS. This way, it provides a thread-like programming style, which is attractive from a developer’s perspective, although different from conventional multi-threading, in the sense that proto-threads do not have any dedicated memory allocation and all processes share a common stack.

A useful characteristic of Contiki OS, which is of high relevance for evaluating the CS-based scheme proposed herein, is a software-based mechanism for profiling communication and computation power consumption of embedded devices. It is further noted that, in this work, we use the latest version of the OS, namely Contiki-NG. In this major upgrade of the OS, the overall code structure was revised and optimized with new configurations and a thorough cleanup of the code base, thus minimizing the final binary size.

#### 3.2.2. Network Stack

**Figure 7:**
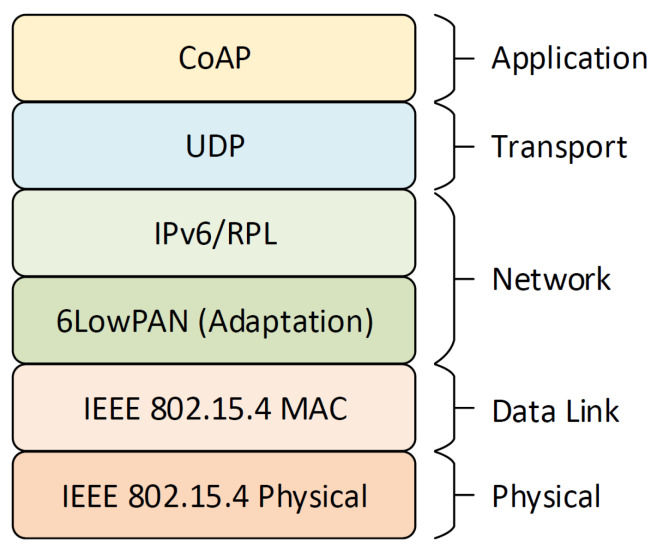
Protocol stack.

The protocol stack architecture used in this work is in accordance with the Internet Engineering Task Force (IETF) recommended stack, as illustrated in Figure 7. The protocol layers are briefly described in the next subsections.

##### IEEE 802.15.4

IEEE 802.15.4 is a standard, which defines both the physical and MAC layer for Low Power Wide Area Networks (LPWANs). The standard operates in both 2.4 GHz and sub-GHz frequency ranges. It was designed by bearing the following requirements in mind: very low complexity, Carrier Sense Multiple Access-Collision Avoidance (CSMA-CA) support, channel hopping, multi-node networks, ultra-low power consumption, low cost, and low data rate. It defines a maximum data rate of 250 kbits/s, depending on the modulation scheme and frequency band selected.

As of Contiki-NG, the implementation offers essentially two different choices for the MAC layer (plus one experimental for BLE radio), namely CSMA (non-beacon-enabled mode, which uses CSMA on always-on radios) and TSCH (Time Slotted Channel Hopping).

##### 6LoWPAN

IPv6 over Low Power Wireless Personal Area Networks (6LoWPAN) defines the standard for IPv6 communication over the physical and MAC layers provided by IEEE 802.15.4. 6LoWPAN acts as an adaptation layer that handles the constraints of the physical layer for providing end-to-end native IPv6 connectivity between a low-power device and any other IPv6 network, including direct connectivity to the Internet. The most profound characteristics of 6LoWPAN are: (i) fragmentation and reassembly of IPv6 packets for supporting the IPv6 minimum Maximum Transmission Unit (MTU) of 1280 bytes; (ii) header compression; (iii) address auto-configuration; and (iv) multicast support (not natively supported by IEEE 802.15.4). In addition, mesh routing is optimized through the RPL (Routing Protocol for Low-power and lossy networks) that provides a mechanism for disseminating information over the dynamic network topology by forming a Destination Oriented Directed Acyclic Graph (DODAG) between the nodes, with the border router (sink node) being the graph’s root.

Contiki-NG achieves full IPv6 compliance through uIP6 implementation [47]. The uIP stack has minimal memory requirements that are satisfied by adopting several strict design choices (e.g., a single packet buffer).

##### CoAP

The Constrained Application Protocol (CoAP) [48] is a RESTful transfer protocol tailored to resource-constrained Internet devices. CoAP follows a request/response interaction model between application endpoints, supports built-in service and resource discovery and can be easily interfaced with HTTP, since it includes key concepts of the Web, such as URIs and Internet media types. It meets specialized requirements, such as multicast support and very low overhead. Since it was originally designed to run on unreliable UDP transport, it integrates a reliability mechanism for managing lost packets at the application layer. Apart from the synchronous request/response mechanism, CoAP supports an asynchronous notifications’ mechanism, named as OBSERVE, that is commonly used for sensor data collection. Finally, it integrates a mechanism, enabled by the Block option, that provides a minimal way to transfer larger representations in a block-wise fashion, for avoiding fragmentation in lower layers. Contiki-NG natively supports CoAP through a refactored implementation of the Erbium library.

#### 3.2.3. Energy Profiling

Energy consumption of a hardware platform running Contiki can be estimated by utilizing the software-based online energy profiling module Energest [49]. Energest employs power state tracking by recording the amount of time (as provided by the platform’s real-time clock) the device spends in different states. It is implemented as a collection of macros and functions; the macros inform the module on component state change and the functions are used for initializing the module and reporting elapsed time in different states. There are four basic Energest types that track four states, respectively: (a) CPU active mode (CPU); (b) CPU low-power mode (LPM); (c) radio transmission (TRANSMIT); and (d) radio listening (LISTEN). Note that there is no separate type for receiving a packet, thus reception is included in LISTEN type.

The consumed power for each mode is calculated by,

(10) $$\mathrm{Consumed}\mathrm{Power}=\frac{\mathrm{Energest}\_\mathrm{Value}\cdot \mathrm{Current}\cdot \mathrm{Voltage}}{\mathrm{RTIMER}\_\mathrm{SECOND}\cdot \mathrm{Runtime}}\;,$$

where Energest_Value is the value returned by Energest (provided in CPU ticks), Current denotes the average current consumption for the mode under consideration (it is hardware-specific and can be retrieved from each hardware component’s datasheet), Voltage is the operating voltage, RTIMER_SECOND is the number of CPU ticks per second for the Contiki RTIMER, and Runtime is the time interval between two Energest tracking points. In Table 1, we report the above values for TI CC2538 SoC.

### 3.3. Implementation Details

To evaluate the energy efficiency of the CS-based system, as well as to provide a comparison against the LZ77 [21] lossless compression alternative, we utilize a simple experimental setup, which consists of a RE-Mote that performs data collection, encoding and transmission, and a gateway that receives and decodes the collected data. The gateway is built by attaching a RE-Mote, which plays the role of the 6LoWPAN border-router, to a PC, which acts as the host. IPv6 traffic between the host and the border router is bridged using Serial Line Internet Protocol (SLIP), provided by tunslip6 utility.

**Figure 8:**
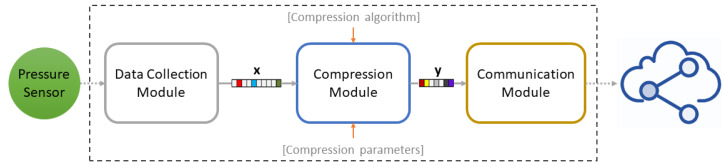
Edge device software layout.

Figure 8 illustrates the software running on the data collection and compression node, which has been implemented in Contiki-NG and consists of the following three distinct modules:

- The Data Collection Module is responsible for the sensor data collection. It periodically polls the sensor for value and buffers them, until a pre-defined block of values is collected.
- The Compression Module, which applies the selected compression algorithm on the collected sensor data. It receives as input: (i) the buffered sensor values provided by the Data Collection Module; (ii) the compression algorithm (CS or LZ77); and (iii) the compression parameters (i.e., the measurement matrix and compression ratio for CS, and dictionary hash table size for LZ77) and outputs a block of compressed data stored in a buffer.
- The Communication Module (built on the network stack described in Section 3.2.2) receives the output of the Compression Module and sends it to the gateway. More specifically, a CoAP server exposes two CoAP resources for managing the compression and collection of compressed data, one for each alternative, namely CS and LZ77. CoAP asynchronous notifications mechanism, OBSERVE, is used for data collection, while appropriate CoAP POST requests permit compression parameters control, such as dynamic compression ratio for CS.

All data are represented as 4-bytes integers. For interfacing the output of the Data Collection Module with the input of the Compression Module, we adopt a double buffering approach, so that sensor data collection does not block while a buffered block of sensor values is being processed by the Compression Module. Additionally, we enable the Energest module for tracking power states during different phases, namely: (i) compression; and (ii) transmission of compressed data. We stress the fact that we focus on these phases, instead of performing a full energy profiling of the device, since we aim at isolating the effect of the CS methodology related to the device consumption.

On the gateway, we run a CoAP client utilizing the Eclipse Californium library (https://www.eclipse.org/californium/), for controlling the compression ratio and observing the resources exposed by the CoAP server, running on the RE-Mote. After the per-block compressed data have been collected, the original sensor values are reconstructed and stored in a local time series database, remaining available for further processing.

Special consideration is taken for improving the efficiency of both lossy and lossless compression process. To decrease the CS compression execution time, we avoid performing a direct matrix multiplication for calculating CS measurements. Instead, considering the fact that a (partial) Hadamard measurement matrix is used, we first apply the Fast Walsh–Hadamard Transform (FWHT) to the block of sensor values, followed by the appropriate sub-sampling for attaining the selected CR. As a result, the computational complexity of CS compression reduces to O(NlogN). Accordingly, since lossless compression algorithms can be in general extremely resource intensive, thus inappropriate for devices with low capabilities, we choose FastLZ (https://github.com/ariya/FastLZ), a small, portable, and efficient byte-aligned LZ77 implementation. After some code modifications, necessary for eliminating memory allocation and usage problems, we successfully satisfied the constraints imposed by the RE-Mote platform.

## 4. Performance Evaluation

In this section, we evaluate the efficiency of the CS-based mechanism for data compression and transmission in a real smart water network test case and illustrate the execution speedup and energy consumption reduction it offers when compared against a well-established lossless compression method that is widely used in commercial solutions, namely, the LZ77 algorithm. We also quantify the energy savings achieved over the scenario of raw (uncompressed) sensor value transmission. Our experiments reveal that the CS-based mechanism can be tuned to operate at a high compression rate (75%), that offers almost 50% savings in terms of transmission energy compared to LZ77 and almost 75% savings compared to raw sensor value transmission, without compromising the decompression fidelity. In addition, we show that the CS lightweight compression mechanism imposes a substantially lower processing overhead compared to that of LZ77, which is almost constant irrespective of the compression rate selected and translates to reduced computational energy consumption. The statistical significance of our results is validated by means of the Kruskal–Wallis test. Finally, we demonstrate the encryption property that is inherent to CS under the assumption that an adversary has full knowledge of the compressed random measurements, as well as a partial knowledge of the measurement matrix up to a permutation of its rows.

### 4.1. Performance Metrics

We define three performance metrics for evaluating our CS-based system: compression execution time (CET), compression energy consumption (CEC), and transmission energy consumption (TEC). The CET is defined as the time the Compression Module needs for calculating the buffered compressed output after receiving a block of sensor values as input and expresses the computational overhead imposed by the compression algorithm. The CEC is the energy spent for the compression of a block of sensor values, as reported by the Energest CPU type. Finally, TEC is the energy spent by the device’s radio for transmitting the block of compressed measurements, as reported by the Energest TRANSMIT type.

We compared the performance of CS against two well-established alternatives, namely: (i) Lempel–Ziv (LZ77) lossless compression and transmission of sensor value blocks; and (ii) transmission of the raw sensor values, without any compression. In our experiments, each pressure sensor was sampled every 15 min, yielding a total of 9984 pressure values for the testing period. Three different block sizes, *N*, were tested with N∈{64,128,256}, in conjunction with three distinct CS compression ratios (${\mathrm{CR}}_{\mathrm{CS}}$), with ${\mathrm{CR}}_{\mathrm{CS}}\in \left\{25\%,50\%,75\%\right\}$. In terms of LZ77 implementation, we fixed the size of the dictionary hash table to be 1 KB, which we empirically found to be a good compromise between memory efficiency and the compression ratio (${\mathrm{CR}}_{\mathrm{LZ}}$). The experimental setup parameters are summarized in Table 2.

To evaluate the effect of different compression types to the performance metrics defined here, we followed a statistical-based approach. Due to lack of normality in our dataset (as reported by Shapiro–Wilk test), we applied the non-parametric Kruskal–Wallis test, followed by Dunn post-hoc test for pairwise comparisons of compression types, in the case a significant difference in the means exists. It is noted that compression type takes values in the set {LZ77, CS-25%, CS-50%, CS-75%}, when considering CET and CEC. In the case of TEC, the compression type belongs to the set {Raw, LZ77, CS-25%, CS-50%, CS-75%}, where the value ”Raw” corresponds to the scenario of raw sensor value transmission, without applying any compression beforehand. The level of significance for all tests was set at p<0.01.

### 4.2. Results

**Figure 9:**
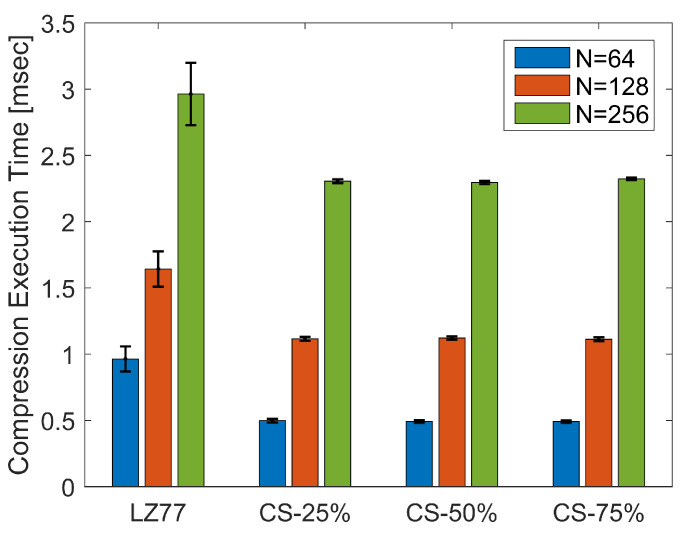
Average and standard deviation of CET, over the total number of pressure blocks, for different compression types.

**Figure 10:**
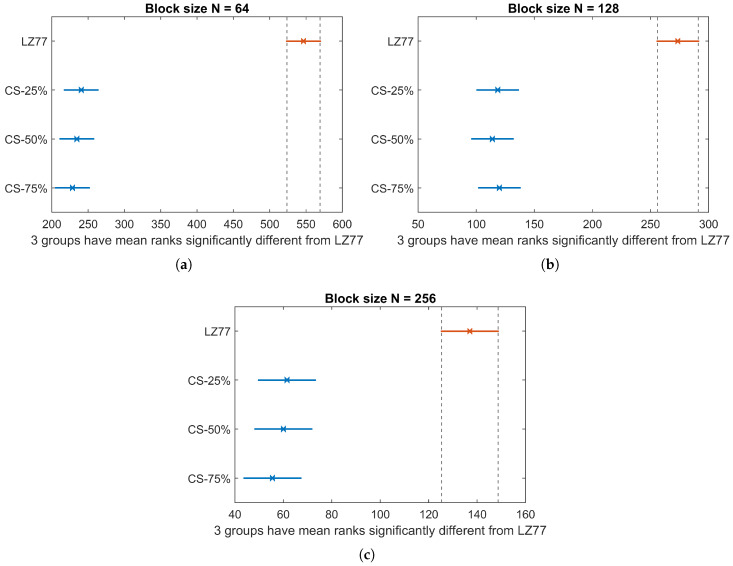
CET Kruskal–Wallis mean ranks and Dunn comparison intervals for different compression types: (**a**) N=64; (**b**) N=128; and (**c**) N=256.

In this section, we present the results in terms of the performance metrics defined in Section 4.1. As a first illustration, Figure 9 shows the CET average and standard deviation (displayed as error bars), over the total number of blocks of pressure values, for the three block sizes *N*, both for lossless (CS) and lossy (LZ77) compression. Kruskal–Wallis test revealed a significant effect of compression type on CET, for all block sizes ($\chi^{2}=483.59$, p<0.001 for N=64, $\chi^{2}=203.39$; p<0.001 for N=128; and $\chi^{2}=116.72$, p<0.001 for N=256). The pairwise multiple-comparison between compression types in Figure 10 shows a significant difference between LZ77 and CS compression for any rate ${\mathrm{CR}}_{\mathrm{CS}}$. However, CET does not differ significantly among CS with different compression rates. This is expected, since, irrespective of ${\mathrm{CR}}_{\mathrm{CS}}$, the CS calculations are dominated by the FWHT applied to the raw sensor values that, as stated before, bears a computational complexity of O(NlogN).

In Table 3, we report the average compression rate ${\mathrm{CR}}_{\mathrm{LZ}}^{\mathrm{av}}$ achieved by LZ77, for the three block sizes. In any case, CS provides a compression speedup of at least 22% over LZ77, even for larger compression rates (75%) than the ones achieved by the lossless algorithm.

**Figure 11:**
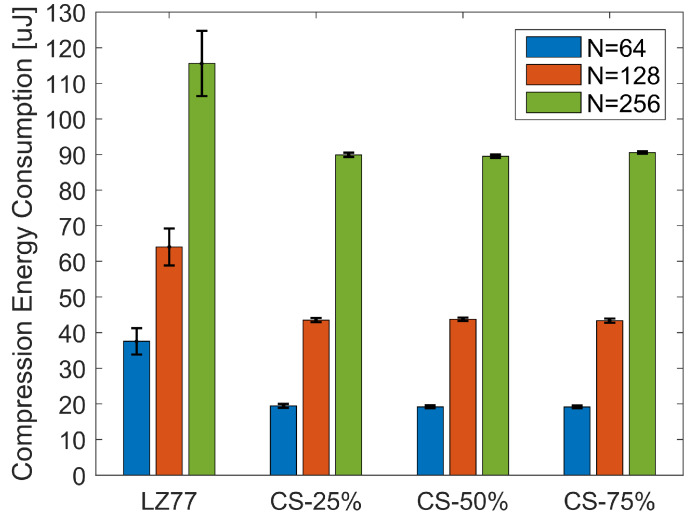
Average and standard deviation of CEC, over the total number of pressure blocks, for different compression types.

**Figure 12:**
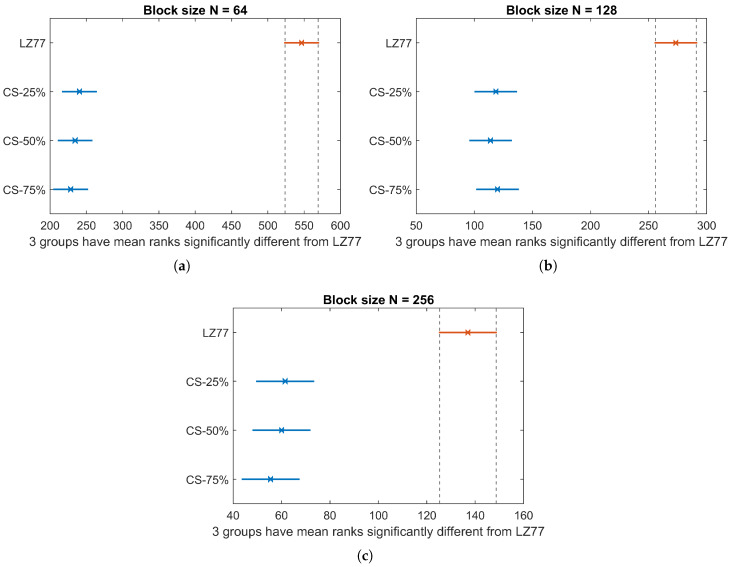
CEC Kruskal–Wallis mean ranks and Dunn comparison intervals for different compression types: (**a**) N=64; (**b**) N=128; and (**c**) N=256.

Figure 11 illustrates the CEC average and standard deviation, over the total number of blocks of pressure values, for the three block sizes *N*. Similar to CET, Kruskal–Wallis analysis (Figure 12) showed that, in terms of CEC, there is a significant difference between lossy and lossless compression ($\chi^{2}=483.59$, p<0.001 for N=64; $\chi^{2}=203.39$, p<0.001 for N=128; and $\chi^{2}=116.72$, p<0.001 for N=256). No significant difference exists in CEC among different compression rates of CS, for a given block size. The average CEC and the savings of CS compression over LZ77 compression are summarized in Table 4. Observe that, even for the smallest block size N=64, we achieve a compression energy saving at the order of 50%.

**Figure 13:**
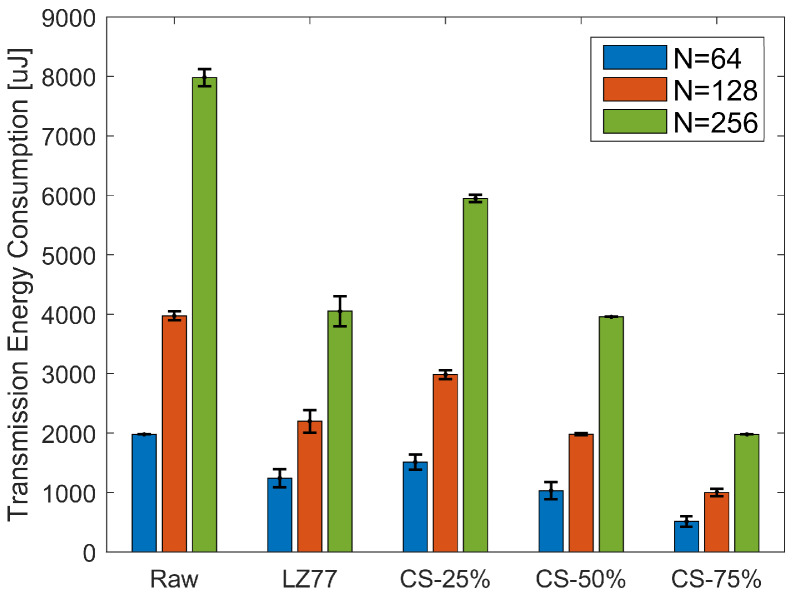
Average and standard deviation of TEC, over the total number of pressure blocks, for different compression types.

**Figure 14:**
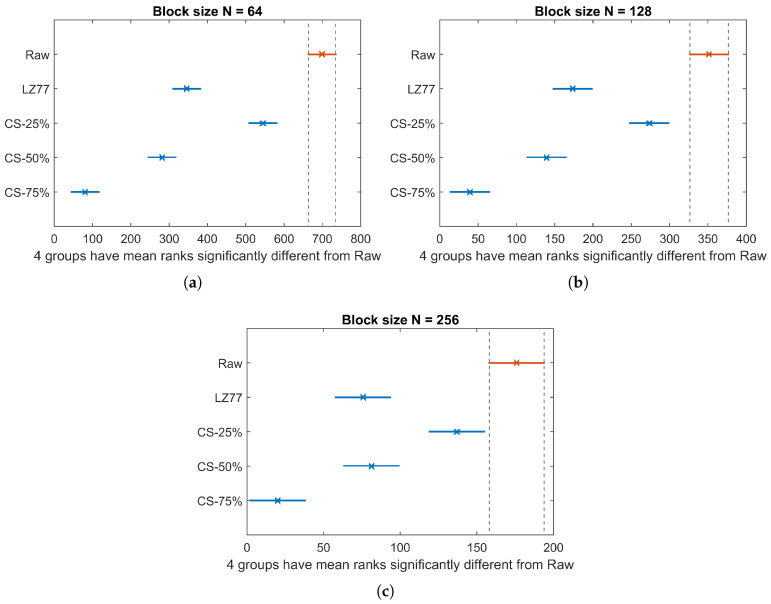
TEC Kruskal–Wallis mean ranks and Dunn comparison intervals for different compression types: (**a**) N=64; (**b**) N=128; and (**c**) N=256.

Figure 13 depicts the TEC average and standard deviation, over the total number of blocks of pressure values, for all three block sizes. Here, the energy consumption for raw sensor value transmission is labeled as “Raw” and corresponds to the worst case in terms of transmission energy cost. According to Kruskal–Wallis test, a significant effect of compression type on TEC exists for all block sizes ($\chi^{2}=716.45$, p<0.001 for N=64; $\chi^{2}=363.95$, p<0.001 for N=128; and $\chi^{2}\;=\;178.71$, p<0.001 for N=256). The multiple-comparison between compression types (Figure 14), shows a significant difference between any compression type pair, apart from the pair {LZ77, CS-50%}, whose elements share an almost equal compression rate (see to Table 3). For all values of *N*, TEC decreases as the CR increases, since less packets need to be transmitted. Finally, in Table 5, we present the total energy consumption savings (by summing CEC and TEC averages) achieved by different compression types, against the energy spent for the transmission of raw sensor values. Observe that in all cases, a larger block size translates to better energy efficiency. This is more profound in the case of LZ77, since the algorithm’s compression efficiency improves as the number of long, repetitive words in the input data increases. Additionally, the overhead imposed by the compression algorithm (and consequently the CEC) is substantially small, so the reported total energy savings primarily result from the gain due to transmitting less data. Thus, CS can achieve significant savings compared to LZ77, if a high CR is selected. Although someone could argue that this could in general compromise the fidelity of CS decompression, we showed that the data used in this application can be accurately reconstructed, even for ${\mathrm{CR}}_{\mathrm{CS}}$ as high as 75% (see Figure 4 and Figure 5).

**Figure 15:**
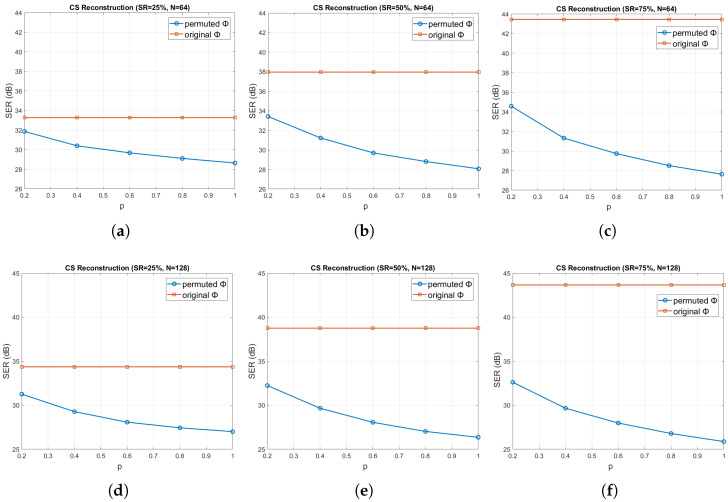

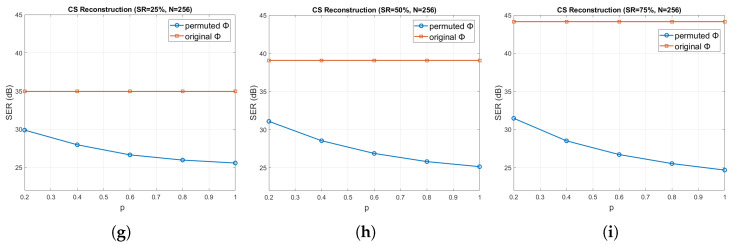
CS reconstruction error in terms of SER (dB) averaged over all streams, for the original and permuted Φ and for SR∈{25%,50%,75%}, as a function of p(%): (**a**–**c**) N=64; (**d**–**f**) N=128; and (**g**–**i**) N=256.

As a last experiment, we demonstrated the weak encryption capability of CS. Specifically, as described in Section 2.3, an adversary has access to the true measurement vector y, whereas the measurement matrix Φ is decrypted up to a permutation of a percentage *p* of its rows, with p∈[0.2,0.4,0.6,0.8,1]. Figure 15 shows the reconstruction error, in terms of the achieved SER (in dB) averaged over all the pressure sensors, for sliding windows of length N∈{64,128,256}, as a function of *p*, for the three sampling ratios SR∈{25%,50%,75%} (or, equivalently, compression ratios CR∈{75%,50%,25%}). Clearly, the reconstruction accuracy deteriorates dramatically, as *p* increases, for all the window lengths and sampling ratios, which verifies the weak encryption capability of CS. The difference in performance between the original and permuted Φ especially increases as the sampling ratio and window length increase. Furthermore, the larger is the window length *N* and the smaller is the CR (i.e., the higher is the SR), the better is the reconstruction performance (i.e., higher SER), as expected.

## 5. Conclusions and Future Work

This study demonstrated the execution and energy efficiency of a CS-based system for smart water network infrastructures equipped with sensing devices with possibly limited power and computational resources. More specifically, our implementation on real hardware revealed a significant reduction of the average execution time up to approximately 50%, when compared against a well-established lossless compression method that is also used in commercial solutions, namely the fast Lempel–Ziv (LZ77) algorithm. Furthermore, our performance evaluation, by varying the sampling ratio and the sliding window length, showed that a CS-based design enables savings of the data compression energy consumption as high as 50% compared with LZ77. Regarding the energy consumed for data transmission, CS can achieve significant savings compared to LZ77 by selecting a high compression ratio, without compromising reconstruction fidelity.

In addition, we successfully demonstrated the weak encryption property of CS, in the case when an adversary has full access to the generated random measurements, but the knowledge of the random measurement matrix is up to a permutation of its rows. Specifically, the experimental results show that, by permuting even 20% of the rows of the measurement matrix, an adversary is not capable of recovering accurately the original sensor stream samples. This is especially important for the design of low-cost smart water monitoring platforms, since no additional software or hardware encryption modules are required.

In the current work, our study primarily focused on the sensing side of smart water network. Nevertheless, in real-time applications, we are also interested in achieving accurate and fast decision making at the side of the control center. For this, we will investigate the effects of the measurement matrix type, as well as of the CS reconstruction algorithm, in the overall system performance, in terms of fast reconstruction while aiming at accurately detecting abnormal events. Furthermore, the weak encryption enabled by CS may not suffice in cases when higher privacy and security standards are required. To this end, we will investigate the combination of a CS scheme with quantum encryption mechanisms towards increasing our system’s security while maintaining the computation cost for edge encryption low enough for smart water networks with limited resources.

## Figures and Tables

**Table 1:** Energest-related values for TI CC2538 SoC.

Variable		Value
RTIMER_SECOND		32,768 ticks
Voltage		3 V
Current	CPU	13 mA
	LPM	0.6 mA
	TRANSMIT (0 dBm)	24 mA
	LISTEN (−100 dBm)	24 mA

**Table 2:** Experimental setup summary.

Parameter	Value
Sensor sampling frequency	1 sample every 15 min
Total number of pressure values in the monitored period	9984
Block size *N*	{64,128,256}
CS compression ratio ${\mathrm{CR}}_{\mathrm{CS}}$	{25%,50%,75%}
Measurement matrix Φ	Hadamard
LZ77 dictionary hash table size	1 KB
Network stack	CoAP + UDP + IPv6 + 6LoWPAN
2]*Physical and MAC layer	Non-beacon-enabled CSMA,
	IEEE 802.15.4
TX power	0 dBm
RF channel	26 (2480 MHz)

**Table 3:** Compression rates for LZ77.

*N*	64	128	256
${\mathrm{CR}}_{\mathrm{LZ}}^{\mathrm{av}}$	44.81%	48.08%	50.04%

**Table 4:** Average CEC and CEC savings of CS compared to LZ77.

*N*	Average CEC [uJ]		CEC Savings
	LZ77	CS (any CR)	
64	38	19	50%
128	64	44	31.25%
256	115	89	22.61%

**Table 5:** Total energy consumption savings compared to raw sensor value transmission.

*N*	LZ77	${\mathrm{CS}}_{\mathrm{CR}}$		
		CR = 25%	CR = 50%	CR = 75%
64	35.43%	22.73%	46.97%	73.07%
128	43.06%	23.82%	49.04%	73.75%
256	47.80%	24.73%	49.28%	74.07%

## Abbreviations

The following abbreviations are used in this manuscript:

WDN	Water Distribution Network
ICT	Information Communication Technologies
CS	Compressive Sensing
SR	Sampling Rate
CR	Compression Rate
STFT	Short-Time Fourier Transform
SER	Signal-to-Error Ratio
IoT	Internet-of-Things
SoC	System on Chip
RF	Radio Frequency
I2C	Inter-integrated Circuit
SPI	Serial Peripheral Interface
UART	Universal Asynchronous Receiver Transmitter
IETF	Internet Engineering Task Force
LPWAN	Low Power Wide Area Network
CSMA-CA	Carrier Sense Multiple Access-Collision Avoidance
TSCH	Time Slotted Channel Hopping
MTU	Maximum Transmission Unit
LoWPAN	Low Power Wireless Personal Area Network
DODAG	Destination Oriented Directed Acyclic Graph
CoAP	Constrained Application Protocol
FWHT	Fast Walsh-Hadamard Transform
CET	Compression Execution Time
CEC	Compression Energy Consumption
TEC	Transmission Energy Consumption
